# Immune Cell-specific Genetic Regulation in Graves’ Disease: An Integrative Mendelian Randomization Study

**DOI:** 10.2174/0118715303437190260604115140

**Published:** 2026-06-22

**Authors:** Shixian Cui, Qingyang Liu, Tianshu Gao

**Affiliations:** 1 Liaoning University of Traditional Chinese Medicine, Shenyang, Liaoning, China;; 2 Department of Endocrinology, Liaoning University of Traditional Chinese Medicine Affiliated Hospital, Shenyang, Liaoning, China

**Keywords:** Graves’ disease, Mendelian randomization, single-cell eQTL, genome-wide association study, immune cell-specific gene expression, bayesian

## Abstract

**Introduction:**

Graves’ disease (GD) is an autoimmune form of hyperthyroidism. We integrated GD GWAS summary statistics with immune-cell eQTLs to prioritize cell-type–specific genes with potential relevance to GD susceptibility.

**Methods:**

We analyzed European-ancestry GD GWAS summary statistics from FinnGen Release 12 together with cis-eQTLs from 14 peripheral immune-cell subsets in the OneK1K single-cell resource (982 donors). We prioritized associations using SMR and HEIDI filtering, and Bayesian colocalization (COLOC) analysis was then performed to assess whether eQTL and GD association signals were likely to share a causal variant. Differential expression analysis using GSE71956 was used as additional transcriptomic support.

**Results:**

After harmonization, 5,048 instrument–gene pairs were retained. MR identified 173 non-redundant genes (FDR < 0.05); SMR plus HEIDI retained 159 genes across the 14 immune subsets. COLOC highlighted 33 high-confidence colocalized signals (PPH4 > 0.90), including ARID5B in CD4^+^ naïve/central memory T cells, PRSS16 in CD4^+^ effector-memory T cells, HIST1H2BI in CD8^+^ effector-memory T cells, and FCRL3 in NK cells. In GSE71956, several prioritized genes showed differential expression, and ARID5B was expressed at lower levels in GD samples than in controls.

**Discussion:**

Concordant evidence across methods supports these signals, but residual pleiotropy and the European-only design remain important caveats. Differences from prior reports may reflect cell-type context and disease stage.

**Conclusion:**

Immune subset–resolved eQTL–GWAS integration points to specific genes and pathways that may contribute to GD susceptibility and helps narrow candidates for follow-up studies.

## 1 INTRODUCTION

Graves’ disease (GD) is an autoimmune form of hyperthyroidism. The disease is primarily driven by autoantibodies against the thyroid-stimulating hormone receptor, which stimulate the receptor and promote excessive thyroid hormone production. Patients commonly present with symptoms related to increased metabolic activity, including palpitations, irritability, weight loss, heat intolerance, and excessive sweating [1]. Some patients may also exhibit ocular symptoms such as exophthalmos, diplopia, and eyelid retraction, significantly impacting quality of life [2]. Although genetic susceptibility, immune dysregulation, and endocrine abnormalities have all been implicated in GD, the mechanisms linking genetic risk variants to immune cell dysfunction remain incompletely understood.

With advances in high-throughput sequencing and bioinformatic analysis, GWAS, transcriptomic profiling, and epigenomic resources have increasingly been used to investigate the genetic basis of autoimmune diseases. However, several challenges persist in GD research. GWAS-identified risk loci often lack sufficient functional annotation, and most GD-associated risk single nucleotide polymorphisms (SNPs) currently have unclear biological roles. Due to immune cell heterogeneity, traditional bulk peripheral blood analyses cannot reveal the specific roles of different immune cell subsets in GD pathogenesis. Additionally, there is a shortage of systematic approaches effectively integrating GWAS data, transcriptional regulation, and functional validation to accurately infer the causality of critical pathogenic genes.

Here, we leveraged cell-type-specific expression quantitative trait locus (eQTL) data supported by single-cell profiling to identify immune cell-specific pathogenic genes in Graves’ disease [3]. Furthermore, Mendelian Randomization (MR) methods help circumvent confounding factors typically encountered in observational studies, thereby establishing more reliable causal inferences between pathogenic genes and disease risks [4]. Integrating GWAS data with immune cell-specific eQTLs may help prioritize genes and cellular contexts that are relevant to GD susceptibility.

This study aims to evaluate the impact of immune cell-specific gene expression on GD risk. We integrated publicly available GD GWAS data with single-cell eQTL data using the summary data-based Mendelian Randomization (SMR) approach, aiming to identify key functional genes potentially causally associated with GD risk from an immune cell perspective. Additionally, differential expression analyses using bulk RNA-seq data from peripheral blood and specific immune cell types were conducted to further support the functional roles of these key genes in GD pathogenesis. By combining genetic association, regulatory evidence, and expression-level validation, this study sought to refine candidate genes and pathways involved in GD immunopathogenesis.

## 2 MATERIALS AND METHODS

This Mendelian randomization study investigated the potential causal relationships between gene expression in specific immune cells and Graves’ disease by integrating eQTL data from the OneK1K resource [5] with genome-wide association study (GWAS) data for Graves’ disease obtained from the GWAS Catalog [6]. Additionally, bulk RNA sequencing (RNA-Seq) datasets from the Gene Expression Omnibus (GEO) [7] were utilized for differential gene expression analysis to identify genes differentially expressed in Graves’ disease. An overview of the study design is illustrated in Fig. (**1**).

### 2.1 Sources of Single-cell RNA eQTL Data

The eQTL data used in this study were derived from the OneK1K cohort, which included 982 donors and 1.27 million peripheral blood mononuclear cells (PBMCs). A total of 26,597 independent cis-eQTLs were identified across 14 immune cell types, including:

CD4^+^ naïve and central memory T cells (CD4NC)

CD4^+^ T cells with effector memory or central memory phenotypes (CD4ET)

CD4^+^ T cells expressing SOX4 (CD4SOX4)

CD8^+^ T cells with effector memory phenotypes (CD8ET)

CD8^+^ naïve and central memory T cells (CD8NC)

CD8^+^ T cells expressing S100B (CD8S100B)

Natural killer (NK) cells

NK-recruited cells (NKR)

Plasma cells

Memory B cells (Bmem)

Immature and naïve B cells (Bin)

Classical monocytes (MONOc)

Non-classical monocytes (MONOnc)

Dendritic cells (DC)

### 2.2 Sources of Outcome Data

Graves’ disease GWAS data were obtained from the GWAS Catalog(FinnGen public results, Release 12; endpoint: E4_GRAVES_STRICT [graves_strict]; accessed on 2025-04-06), comprising 3,962 cases and 496,386 controls of European ancestry [8].

### 2.3 Sources of Bulk RNA-Seq Data

The GEO dataset GSE71956 was used for expression-level validation. This dataset contains gene expression profiles of CD4^+^ and CD8^+^ T cells from patients with GD and healthy controls [9].

### 2.4 Instrument Variable Selection

In Mendelian randomization (MR) analysis, cis-eQTL variants were used as instrumental variables (IVs) to evaluate gene expression. The cis-region was defined as ±2 Mb around the probe. Only genes with at least one cis-eQTL (*P* < 5.0 × 10^−8^) were considered. SNPs with allele frequency differences > 0.2 between eQTL and GWAS datasets were excluded, allowing up to 5% SNP exclusion based on allele frequency discrepancies [10]. The strength of genetic instruments was assessed using the F-statistic (F = β^2^/SE^2^), and SNPs with F < 10 were excluded [11]. Data harmonization and instrument filtering were performed using SMR software (version 1.3.1, https://yanglab.westlake.edu.cn/software/smr/#Download) [12-14].

### 2.5 Mendelian Randomization Analysis

In the MR analysis, we used the TwoSampleMR package for data processing [15]. Instrumental variables (IVs) were selected based on significant associations with cis-eQTL variants. For cis-eQTLs associated with a single SNP, the Wald ratio method was used to estimate causal effects [16]. For genes with multiple cis-eQTLs, the inverse-variance weighted (IVW) method was applied to aggregate the effects [17]. All results were corrected for false discovery rate (FDR) to control for errors due to multiple comparisons [18].

### 2.6 SMR Analysis

To infer the causal relationship between gene expression and Graves' disease phenotypes, we integrated GWAS and eQTL summary data using the SMR method (version 1.3.1) in the SMR software described above.

First, we matched SNPs between the GWAS and eQTL datasets, ensuring they were fully aligned in genomic position. Then, we applied the SMR method to compute the test statistics for each matched SNP and adjusted the p-values using the Benjamini-Hochberg procedure to control for the false discovery rate (FDR). Additionally, we performed the HEIDI test to differentiate between causal effects and colocalization effects, ensuring the reliability of the analysis results.

### 2.7 Colocalization Analysis

To evaluate whether there is a shared causal variant between genes and Graves' disease, we performed colocalization analysis using the COLOC R package. Prior settings (using the default values in the coloc package):p_1_ = 1 × 10^−4^ – prior probability that any given SNP is an eQTL,p_2_ = 1 × 10^−4^ – prior probability that any given SNP is associated with Graves’ disease risk,p_12_ = 1 × 10^−5^ – prior probability that any given SNP influences both traits [19].

In this analysis, we tested five hypotheses and used posterior probability (PPH4) to measure the support for each hypothesis:

H0 (No causal variant): This hypothesis assumes there is no shared causal variant between the gene and the disease. If PPH4 < 0.9, we reject the hypothesis and conclude that there is no shared causal variant between the gene and the disease.

H1 (Causal variant only in the gene): This hypothesis assumes that only the gene has a causal variant, and that the disease and gene do not share a causal variant. If PPH4 > 0.9 for the gene and the P-value is small, this hypothesis is supported.

H2 (Causal variant only in the disease): This hypothesis assumes that only the disease has a causal variant, with no shared causal variant between the gene and the disease. If the disease’s PPH4 > 0.9 and the P-value is small, this hypothesis is supported.

H3 (Two independent causal variants): This hypothesis assumes that the gene and disease each have independent causal variants, without a shared causal variant. If both the gene and disease have relatively high PPH4 values but do not meet the criteria for shared causal variants, this hypothesis is supported.

H4 (Shared causal variant): This hypothesis assumes that the gene and disease share a causal variant. If PPH4 > 0.9, then we conclude that the gene and disease share a causal variant, supporting this hypothesis [20].

All tests of these hypotheses were based on posterior probabilities (PPH4) calculated by the COLOC R package. To control for errors due to multiple hypothesis testing, all P-values were adjusted using FDR correction. A PPH4 > 0.9 was considered strong evidence supporting the hypothesis, whereas lower PPH4 values were considered to provide insufficient support.

### 2.8 Tissue Expression Analysis

Tissue-specific expression patterns of the prioritised genes were examined using FUMA [21]. The input gene set consisted of the prioritised genes identified from the MR, SMR/HEIDI, and colocalisation analyses. Tissue expression profiles were derived from the Genotype-Tissue Expression (GTEx) project, and gene expression levels across human tissues were visualised as a heatmap using the default FUMA settings [22].

### 2.9 Differential Gene Expression Analysis

In this study, we used the limma method to identify differentially expressed genes (DEGs) between Graves' disease patients and healthy controls. Prior to DEG analysis, all gene expression data were normalized, and the limma package was applied to transform the data for RNA-seq analysis. We selected genes with a P-value less than 0.05 and a log_2_(fold change) greater than 1.5 as DEGs [23]. All P-values were adjusted using the false discovery rate (FDR) to control for errors due to multiple testing.

After identifying DEGs, GO and KEGG enrichment analyses were performed using the clusterProfiler package [24]. All pathway analyses were also adjusted using the Benjamini-Hochberg procedure to control for the false discovery rate, with adjusted *P*-values (*p* < 0.05).

## 3 RESULTS

### 3.1 Mendelian Randomization

After harmonizing 26 634 cis-eQTLs with GD GWAS summary statistics and removing ambiguous SNPs, we retained 5 048 instrument–gene pairs covering 4 172 unique probes.

Using the Wald ratio (single-SNP) or IVW model (multi-SNP), and applying a Benjamini-Hochberg FDR < 0.05, 173 non-redundant genes showed putative causal effects on GD (Figure **S1**–**S4**, Supplementary Material **1**). Among them, 110 genes increased GD risk (positive β), whereas 63 genes decreased risk (negative β).

Steiger filtering confirmed that for all 173 genes the direction of causality was gene-expression → disease, not *vice-versa* (Supplementary Material **2** and **3**).

Among the 173 MR-nominated genes, we first applied the summary-data-based Mendelian randomization (SMR) test to harmonized cis-eQTL and GD-GWAS data, retaining signals with FDR_SMR_< 0.05. We then used the HEIDI outlier test (P_HEIDI_ > 0.05) to remove linkage-driven false positives. This two-step screen yielded 159 non-redundant genes distributed across 14 peripheral immune-cell subsets. The corresponding association landscapes are displayed in Fig. (**2**) (eight panels) and Fig. (**3**) (six panels): each panel depicts the –log_10_ P_SMR_ profile for one cell type, with the dashed line marking the FDR 0.05 threshold and the red dot highlighting the most significant SNP–gene pair.

### 3.2 Colocalisation of SMR/HEIDI-prioritised Signals

To determine whether the SMR/HEIDI-prioritised association signals were driven by a shared causal variant, we performed COLOC colocalisation analysis on the 159 gene–cell-type signals retained after SMR (FDR_SMR < 0.05) and HEIDI filtering (P_HEIDI > 0.05). We considered PPH4 > 0.90 as strong evidence of colocalisation and ultimately identified 33 high-confidence colocalised signals across all 14 immune-cell subsets. Representative examples include ARID5B in CD4^+^ naïve/central memory T cells (top SNP rs6479789; PPH4 ≈ 0.96), PRSS16 in CD4^+^ effector-memory T cells (rs35656932), HIST1H2BI in CD8^+^ effector-memory T cells (rs72839445; PPH4 ≈ 0.995), and FCRL3 in NK cells (rs2210912; PPH4 ≈ 0.90). These colocalisation results further reduced the SMR/HEIDI-prioritized associations to a smaller group of gene–cell-type–variant signals with stronger genetic support. Full COLOC posterior probabilities and top variants are summarised in Supplementary Material **4**.

### 3.3 Tissue Expression Analysis

Tissue-specific expression of the prioritised genes was queried using FUMA. Expression profiles across human tissues were derived from GTEx, and visualised as a tissue-expression heatmap (Fig. **4A**). The input gene set consisted of the prioritised genes identified in the MR/SMR/colocalisation analyses.

### 3.4 Differential Expression of Identified Genes

For the GSE71956 dataset, per-sample boxplots of normalised expression values (Fig. **4B**) were used as a quality-control summary to assess distribution comparability after normalisation and to flag potential outliers. Differential expression between Graves’ disease patients and healthy controls is visualised in the volcano plot (Fig. **4C**) based on limma statistics.In GSE71956, CD4^+^ and CD8^+^ T-cell samples were pooled for an overall expression-level validation, and boxplots were used to visualise between-group differences for selected candidate genes (Fig. **4D**-**E**); notably, ARID5B shows lower expression in Graves’ disease patients compared with controls (Fig. **4D**), consistent with its protective direction inferred from the MR analysis.

### 3.5 GO and KEGG Enrichment

GO analysis of the colocalised gene set revealed strong up-regulation of innate-immune processes—including neutrophil degranulation, neutrophil activation involved in immune response, and antimicrobial humoral defence—as well as vesicle-acidification terms such as proton-exporting V-type ATPase complex (Fig. **5A**).

In KEGG, the up-regulated genes were primarily enriched for classical host-defence pathways (natural-killer-cell–mediated cytotoxicity, Fc-γR–mediated phagocytosis, NOD-like-receptor signalling, phagosome) and infection signatures (*e.g.*, Leishmaniasis, Tuberculosis); (Fig. **5B**).

By contrast, down-regulated genes clustered in ribosome/mitochondrial compartments and stress-response terms (ribosome biogenesis, unfolded-protein response, cellular respiration; Fig. **5C**). KEGG down-regulation centred on oxidative phosphorylation and multiple neurodegenerative disease pathways (Parkinson, Alzheimer, Huntington), together with T-cell signalling modules (T-cell-receptor signalling, Th1/Th2 and Th17 differentiation) (Fig. **5D**).

## 4 DISCUSSION

Before in-depth discussion of the 33 significantly associated genes, we first excluded two categories of genes based on their biological relevance and current research status: histone family genes (such as the HIST1H series), due to their broad involvement in chromatin structural modifications and generally non-specific roles, making their specific roles in GD immunopathology difficult to ascertain; and long non-coding RNAs (such as RP11-367G6.3, CTA-14H9.5, HCG11, RP1-30M3.5, U91328.19, C11orf24, and LINC00240) and pseudogenes, due to insufficient functional annotations and literature support. Consequently, ten genes (ARID5B, ACAP1, SESN3, PRSS16, MMEL1, ZNF322, FCRL3, BTN2A1, HMGN4, ZBTB9) were retained, each having well-defined biological functions in immune regulation and existing research support.

In our research, SMR and bulk RNA-seq analyses from GD patient samples supported a crucial biological function of ARID5B in GD.

Previous fine-mapping work in autoimmune thyroid disease has implicated ARID5B as a susceptibility-related gene [25]. The direction of effect observed in our study differs from that reported in some previous studies. However, evidence from other autoimmune diseases supports the possibility that ARID5B may exert immunomodulatory or protective effects in specific disease contexts. For instance, the long isoform of ARID5B has been reported to suppress TNF-α-induced IL-6 expression in synovial fibroblasts in RA, exerting immunosuppressive effects [26]. Furthermore, a GWAS study in the Chinese Han population reported a negative association between the ARID5B variant rs4948496 and systemic lupus erythematosus (SLE), reinforcing the gene’s potential protective role in certain autoimmune diseases [27].

The ARID5B gene, located on chromosome 10q21.2, is a member of the ARID transcription factor family. ARID family members bind specifically or non-specifically to AT-rich genomic DNA sequences, functioning as epigenetic regulators in chromatin remodeling and gene expression. ARID5B genotype risk alleles and decreased ARID5B expression have also been linked to leukemia relapse [28]. Experimental studies in mouse models have shown that Arid5b overexpression reduces B-cell numbers in the bone marrow, spleen, and peripheral blood and affects B-cell maturation. Although ARID5B-overexpressing B cells showed higher basal B-cell receptor (BCR) signaling and mitochondrial oxygen consumption rates, their proliferative and activation capacities were significantly impaired upon antigen stimulation.

These observations suggest a complex regulatory role for ARID5B in immune homeostasis and response, possibly mediated *via* metabolic and signaling pathways [29]. Further, ARID5B enhances mitochondrial oxidative phosphorylation and anti-apoptotic gene expression (*e.g.*, BCL-2), promoting adaptive NK cell survival and IFN-γ secretion, thus maintaining immune memory functions. Similar regulatory roles are also observed in CD4^+^ T cells, CD8^+^ T cells, and mature B cells [30]. This study focused on human cytomegalovirus (HCMV) infection but also highlights how ARID5B plays multiple roles in regulating immune processes. These findings suggest that ARID5B warrants further investigation in the context of GD immunopathogenesis.

A large-scale GWAS study among Chinese Han individuals identified significant associations between the rs12575636 locus on 11q21 and GD, which were completely linked to the European-reported rs4409785. cis-eQTL analyses further showed close associations between these SNPs and SESN3 expression in CD4^+^ T cells, suggesting SESN3’s role in GD through oxidative stress regulation [31]. Notably, our study similarly identified rs4409785 as a regulator of SESN3 in CD4^+^ T cells, a GD risk factor. SESN3 is a stress-response protein that regulates cellular redox balance and mitigates oxidative stress by lowering ROS levels [32], thereby potentially providing protective effects in immune-related diseases such as GD.

The PRSS16 gene resides within the HLA extended region, highly associated with GD [33]. PRSS16 encodes a thymus-specific serine protease and has been implicated in thymic selection and autoimmune susceptibility [34]. Contrastingly, our findings indicated a positive correlation between PRSS16 expression in peripheral immune cells (including CD4^+^ effector T cells, CD4^+^ naïve T cells, and memory B cells) and GD risk, suggesting a distinct peripheral regulatory function.

An ImmunoChip study identified MMEL1 (rs2843403) as significantly associated with GD and suggested a protective effect [35], contradicting our findings. Furthermore, a large-scale GWAS in the Chinese Han population initially validated MMEL1 as a potential GD risk factor, aligning with our results; however, significance diminished in subsequent validations, possibly due to sample size or population heterogeneity [31].

ZNF322 has been reported to regulate AP-1-related transcriptional activity [36]. Although its role in GD remains unclear, our results suggest that it may represent a candidate for future functional investigation.

A transcriptome-wide association study for systemic lupus erythematosus (SLE) evaluated SLE-associated GWAS loci across various immune cell types and demonstrated allele-specific regulatory effects of ACAP1 near rs61759532. Its risk allele significantly reduced ACAP1 expression, thereby increasing SLE susceptibility [37]. This finding suggests ACAP1 may play a critical role in maintaining immune homeostasis and suppressing SLE development.Although ACAP1 has not been directly linked to GD, these findings support its potential relevance as an immunoregulatory candidate that merits further study.

In recent years, several studies have revealed potential roles of the FCRL3 gene in GD pathogenesis. A UK case-control study including 1056 GD patients and 864 healthy controls demonstrated multiple SNPs related to FCRL3 positively associated with GD. Functional studies suggested these variants might promote autoimmune responses and exacerbate GD severity by influencing B-cell signaling and activation pathways [38], contrasting with the protective effects observed in our study. In a Chinese population, a cross-sectional study involving 436 GD patients and 316 controls identified a protective role for the FCRL3 rs7528684 polymorphism against GD, consistent with our findings [39]. However, another study in the Chinese Han population suggested FCRL3 and its proxy SNP rs7528684 might participate in GD pathogenesis by suppressing B-cell receptor BCR signaling and impairing Tregs function, indicating a positive association [40]. Additionally, a Polish study analyzing peripheral blood samples revealed significantly elevated FCRL3 mRNA expression in GD patients compared to Graves ophthalmopathy patients and healthy individuals, closely correlating with hyperthyroidism but not thyroid antibody levels [41].

FCRL3 belongs to the Fc receptor family and encodes a membrane-bound protein with both immunoreceptor tyrosine-based activation motifs (ITAM) and immunoreceptor tyrosine-based inhibitory motifs (ITIM), providing dual regulatory potential [42]. Research indicates that despite possessing ITAM and ITIM-like sequences, FCRL3 predominantly suppresses BCR signaling by recruiting phosphatases like SHP-1, SHP-2, and SHIP [43].

Reduced B-cell receptor signaling may limit autoreactive B-cell activation in some autoimmune contexts, particularly in diseases characterized by excessive B-cell activation and autoantibody production [44, 45]. However, excessive suppression of B-cell receptor signaling may also impair normal negative selection or apoptosis of autoreactive B cells, thereby disrupting immune tolerance [43].

The roles of BTN2A1, HMGN4, and ZBTB9 in GD remain poorly characterized. Existing studies have mainly focused on their involvement in tumor biology, cell proliferation, apoptosis, and immune escape. Their prioritization in the present analysis suggests possible links to GD susceptibility, but the underlying mechanisms remain uncertain. Future advancements in data resources and methodologies may highlight these genes as novel research targets, contributing to a deeper understanding of the genetic basis and immune regulatory networks in GD.

## 5 STRENGTHS AND LIMITATIONS

First, we integrated multi-layered data, including eQTL, GWAS, and RNA-seq datasets, and utilized MR methods to analyze the causal relationships between gene expression and GD. Analysis of eQTL data across 14 immune cell types elucidated the cell-specific expression patterns of GD-associated genes. Second, we analyzed both the overall effects and the individual contributions of different immune cell types, accounting for the heterogeneity inherent in GD. Third, sufficient gene expression data and bulk RNA-seq data supported the reliability of our results. Finally, combining MR-based prioritization with HEIDI and colocalization analysis reduced the likelihood that the prioritized associations were driven solely by linkage disequilibrium.

Our study also has limitations. First, the analyses were based exclusively on GWAS and eQTL data from European populations, limiting generalizability due to potential genetic heterogeneity across populations. Second, the relatively small eQTL sample sizes might cause missing weak associations or insufficient exploration of rare variants. We applied strict statistical techniques to reduce bias, but small sample sizes could limit comprehensive gene identification.

## CONCLUSION

The main method for our research was the integration of GD GWAS with single-cell eQTLs across 14 immune-cell subsets and validation using bulk RNA-seq, through which we identified genes having possible causal roles—most significantly ARID5B, FCRL3, and SESN3—that linked immune-regulatory and tolerance pathways to GD. These results clearly show what needs to be tested in future studies and point to candidate biomarkers and targets for precision immunotherapy.

## Figures and Tables

**Fig. (1):**
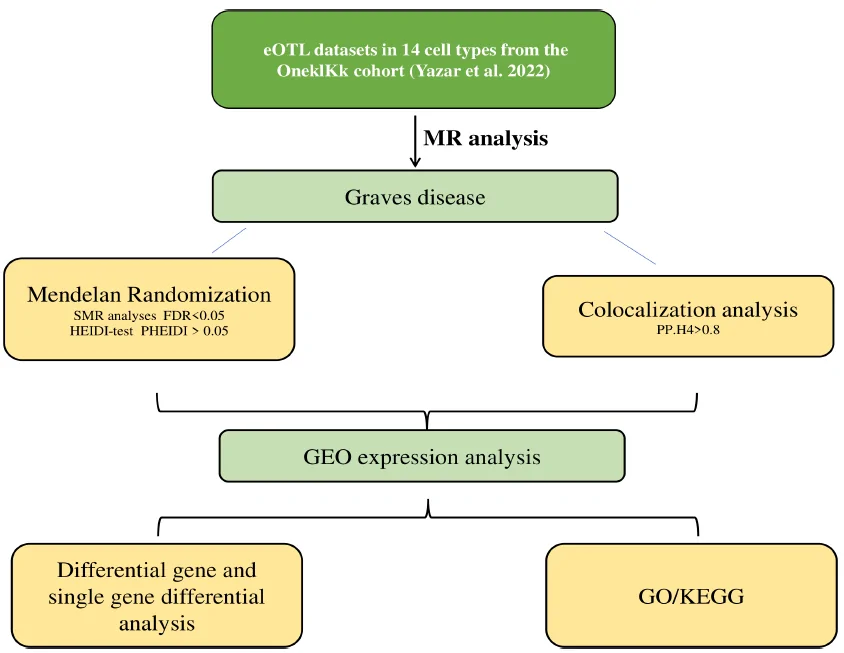
Overview of the study design.

**Fig. (2):**
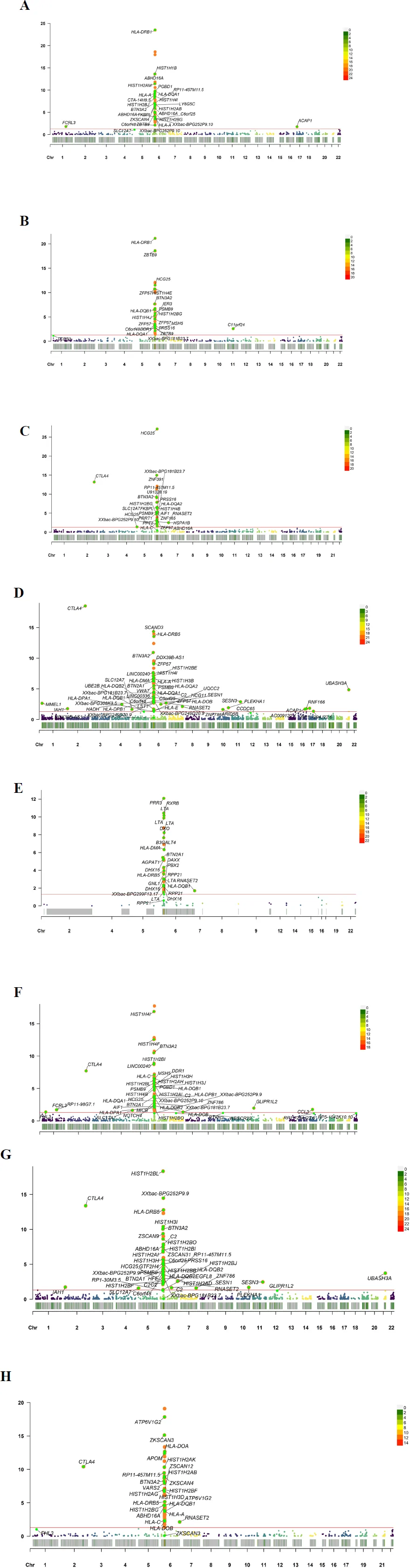
SMR association landscapes for Graves’ disease across eight immune cell types. Each panel (**A–H**): Bin, Bmem, CD4ET, CD4NC, CD4SOX4, CD8ET, CD8NC, CD8S100B) shows the summary-data-based Mendelian randomization (SMR) results for a specific immune cell type. The x-axis denotes chromosomal position, and the y-axis shows −log_10_(P_SMR), representing the strength of association between genetically predicted gene expression and Graves’ disease risk. Each point represents a tested gene (or expression probe) and is positioned according to the genomic location of its top cis-eQTL. The horizontal dashed line indicates the significance threshold after false discovery rate (FDR) correction. Selected genes are annotated for illustration. Colocalisation evidence (COLOC posterior probabilities) is reported separately in Supplementary Material **4**.

**Fig. (3):**
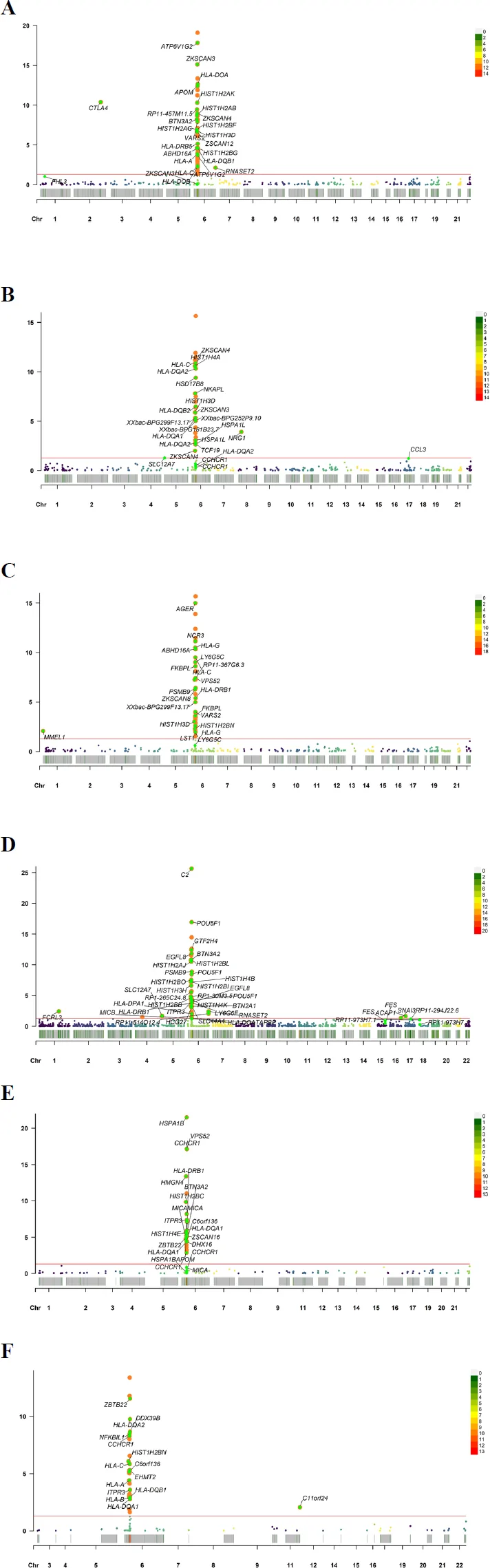
SMR association landscapes for Graves’ disease across additional immune cell types. Each panel (**A-F**): dendritic cells, classical monocytes, non-classical monocytes, NK cells, NKR cells, and plasma cells) displays SMR results for the indicated immune cell type. The x-axis denotes chromosomal position, and the y-axis shows −log_10_(P_SMR). Each point represents a tested gene (or expression probe) based on its top cis-eQTL. The horizontal dashed line marks the FDR-corrected significance threshold. Representative genes are annotated. Colocalisation results are not shown in these plots and are summarised in Supplementary Material **4**.

**Fig. (4):**
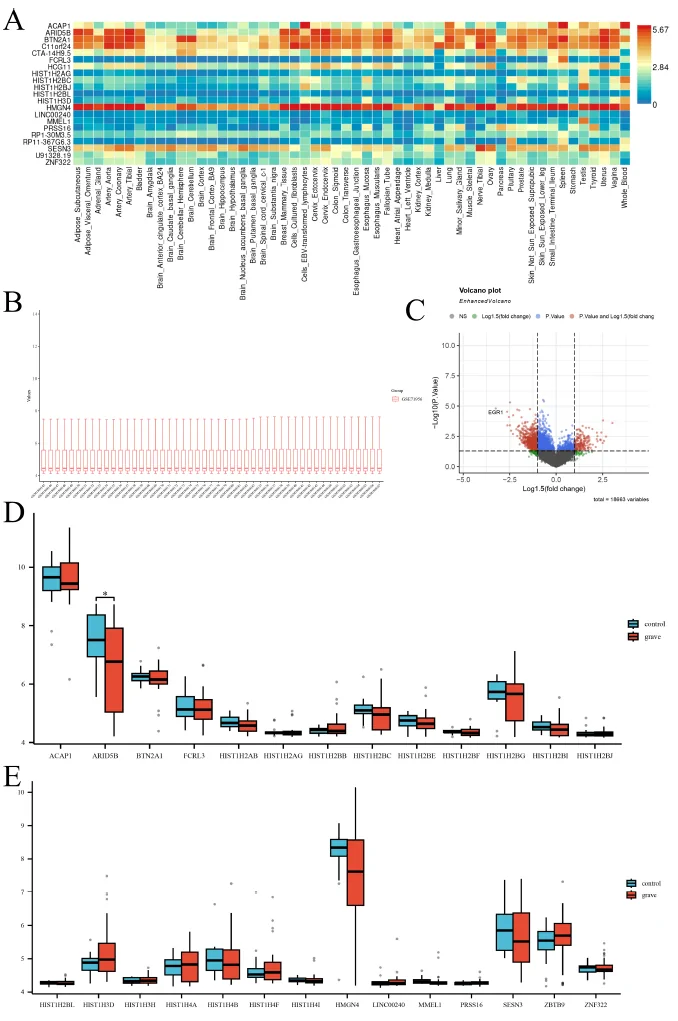
(**A**) Tissue-expression heatmap of prioritised genes across human tissues (rows, genes; columns, tissues) based on GTEx expression queried *via* FUMA; colour indicates normalised expression levels. (**B**) Boxplots of normalised expression values for each sample in the GSE71956 dataset (each box represents one sample), shown to assess distribution comparability after normalisation and to identify potential outliers/technical variation. Samples are ordered and annotated by group: CD4_GD (n=15), CD8_GD (n=16), CD4_con (n=10), and CD8_con (n=8). (**C**) Volcano plot showing differential expression between Graves’ disease patients and healthy controls in GSE71956. The x-axis indicates log2 fold change (log2FC) and the y-axis indicates –log10 (*P* values). Differential expression was assessed using limma; significant DEGs were defined and highlighted using *p* < 0.05 and log2 fold change > 1.5. (**D-E**) Boxplots comparing expression of selected candidate genes between Graves’ disease patients and healthy controls in pooled CD4^+^ and CD8^+^ T-cell samples from GSE71956 (blue, controls; red, Graves’ disease). * indicates statistical significance (*P* < 0.05; limma).

**Fig. (5):**
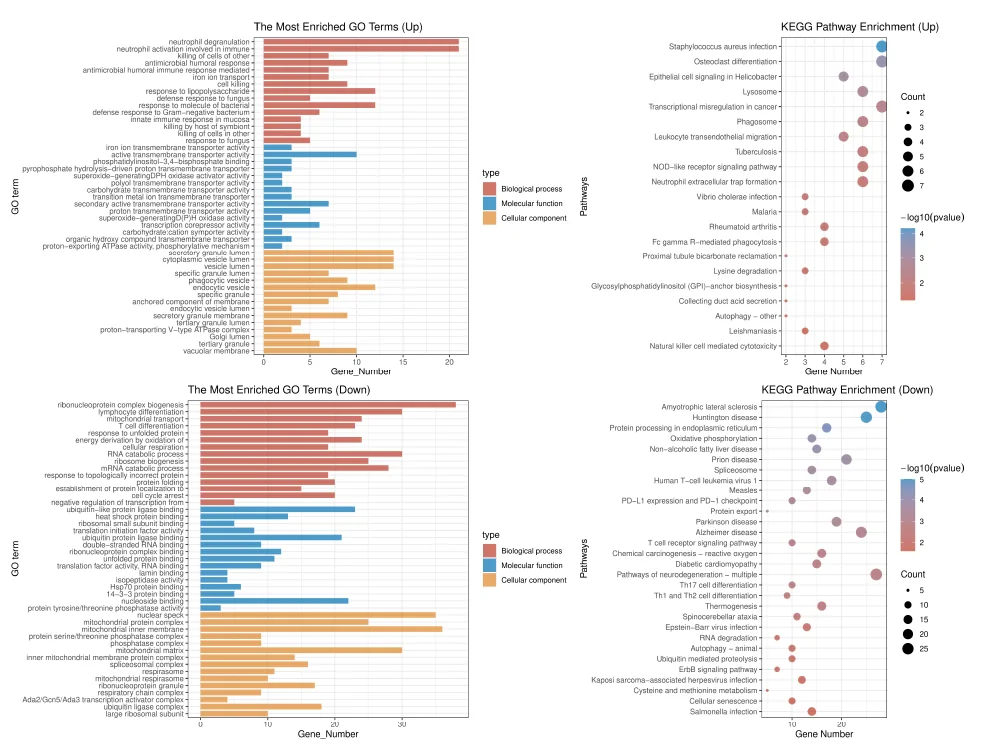
(**A**) The most enriched GO terms (upregulated) in Graves' disease. Bar plot showing the top GO biological process, molecular function, and cellular component terms that are most enriched in the upregulated genes. (**B**) KEGG pathway enrichment (upregulated) for Graves' disease. The scatter plot shows the top enriched KEGG pathways associated with the upregulated genes. The size of the circles represents the gene count, and the color intensity represents the statistical significance (-log10 *p*-value). (**C**) The most enriched GO terms (downregulated) in Graves' disease. Bar plot showing the top GO biological process, molecular function, and cellular component terms that are most enriched in the downregulated genes. (**D**) KEGG pathway enrichment (downregulated) for Graves' disease. The scatter plot shows the top enriched KEGG pathways associated with the downregulated genes. The size of the circles represents the gene count, and the color intensity represents the statistical significance (-log10 p-value).

## Data Availability

Not applicable.

## LIST OF ABBREVIATIONS

Bin: Immature and Naïve B Cells
Bmem: Memory B Cells
BCR: B-Cell Receptor
CD4ET: CD4^+^ T Cells with Effector Memory/Central Memory Phenotypes
CD4NC: CD4^+^ Naïve and Central Memory T Cells
CD4SOX4: CD4^+^ T Cells Expressing SOX4
CD8ET: CD8^+^ T Cells with Effector Memory Phenotypes
CD8NC: CD8^+^ Naïve and Central Memory T Cells
CD8S100B: CD8^+^ T Cells Expressing S100B
DC: Dendritic Cells
DEGs: Differentially Expressed Genes
eQTL: Expression Quantitative Trait Locus
FDR: False Discovery Rate
FC: Fold Change
FUMA: Functional Mapping and Annotation
GD: Graves’ Disease
GEO: Gene Expression Omnibus
GO: Gene Ontology
GWAS: Genome-Wide Association Study
HEIDI: Heterogeneity in Dependent Instruments
HCMV: Human Cytomegalovirus
IV: Instrumental Variable
IVW: Inverse-Variance Weighted
KEGG: Kyoto Encyclopedia of Genes and Genomes
MONOc: Classical Monocytes
MONOnc: Non-Classical Monocytes
MR: Mendelian Randomization
NK: Natural Killer (Cell)
NKR: NK-Recruited Cells
PBMC: Peripheral Blood Mononuclear Cells
RA: Rheumatoid Arthritis
RNA-seq: RNA Sequencing
scRNA-seq: Single-Cell RNA Sequencing
SLE: Systemic Lupus Erythematosus
SMR: Summary Data-Based Mendelian Randomization
SNP: Single-Nucleotide Polymorphism

## STANDARDS OF REPORTING

STROBE-MR guidelines and methodology were followed.
