# Supplementary material for: Immune Cell-specific Genetic Regulation in Graves’ Disease: An Integrative Mendelian Randomization Study
Source: Endocr Metab Immune Disord Drug Targets. 2026 Jun 22;26:E18715303437190. doi: 10.2174/0118715303437190260604115140 (PMC13629157; doi:10.2174/0118715303437190260604115140)
Supplement: Supplementary file 1 [file EMIDDT-26-E18715303437190_SD1.zip › EMIDDT-26-E18715303437190_SD1/BMS-EMIDDT-2025-403-SP.pdf]

Supplementary Material

Immune Cell-Specific Genetic Regulation in Graves' Disease: An Integrative Mendelian Randomization Study

Shixian Cui[1], Qingyang Liu[2,*] and Tianshu Gao[2,*]

[1]*Liaoning University of Traditional Chinese Medicine, Shenyang, Liaoning, China;* [2]*The Department of Endocrinology, Liaoning University of Traditional Chinese Medicine Affiliated Hospital, Shenyang, Liaoning, China*

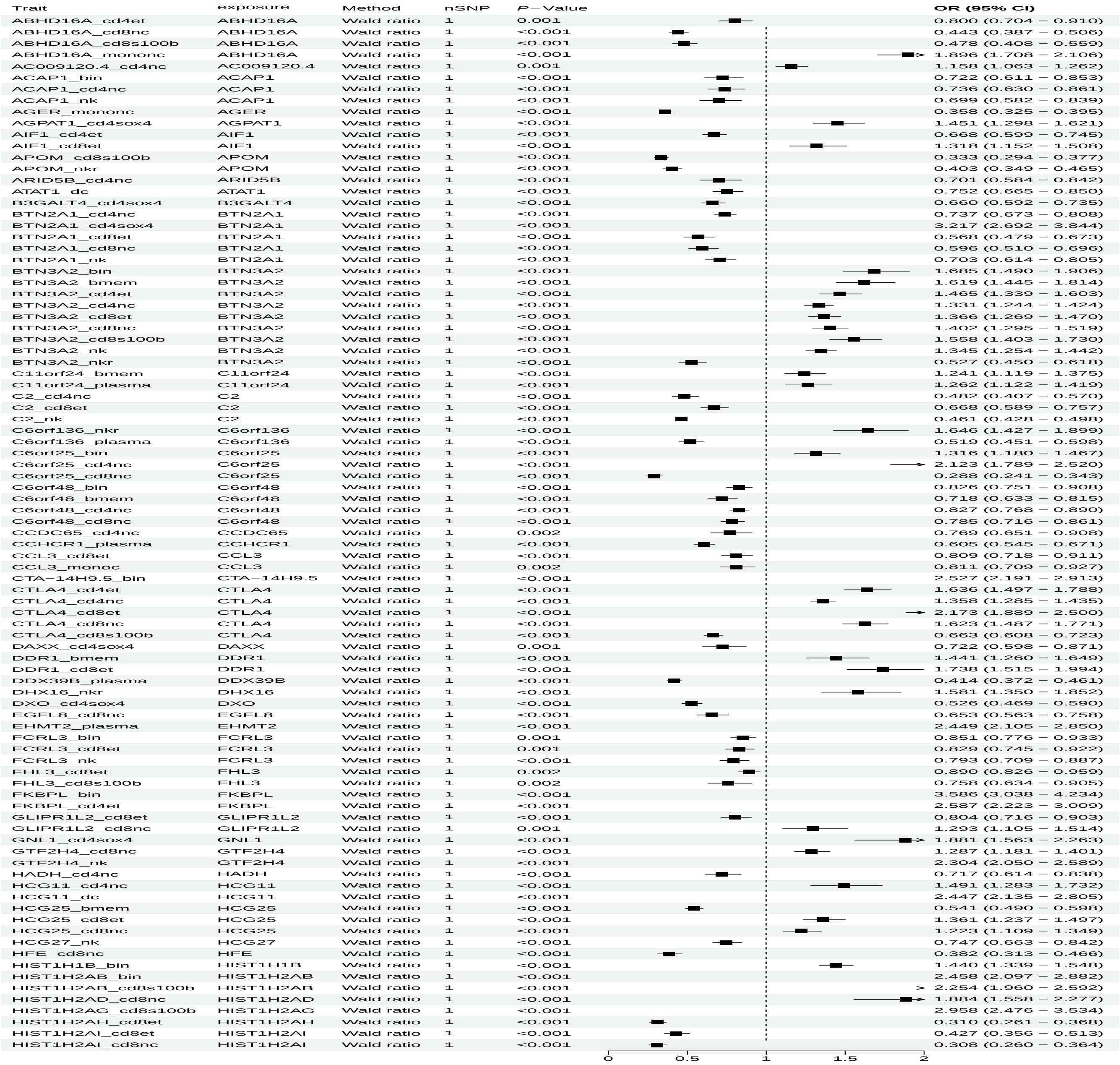

Fig. (S1).

Trait	exposure	Method	nSNP	P−Value	OR (95% CI)
HIST1H2AJ_nk	HIST1H2AJ	Wald ratio	1	<0.001	0.389 (0.338 − 0.447)
HIST1H2AK_cd8s100b	HIST1H2AK	Wald ratio	1	<0.001	2.028 (1.785 − 2.305)
HIST1H2AM_bin	HIST1H2AM	Wald ratio	1	<0.001	0.421 (0.372 − 0.476)
HIST1H2BB_cd8nc	HIST1H2BB	Wald ratio	1	<0.001	1.856 (1.593 − 2.162)
HIST1H2BB_nk	HIST1H2BB	Wald ratio	1	<0.001	0.320 (0.261 − 0.393)
HIST1H2BC_nkr	HIST1H2BC	Wald ratio	1	<0.001	0.451 (0.396 − 0.514)
HIST1H2BE_cd4nc	HIST1H2BE	Wald ratio	1	<0.001	2.543 (2.166 − 2.985)
HIST1H2BF_cd8nc	HIST1H2BF	Wald ratio	1	<0.001	0.690 (0.595 − 0.801)
HIST1H2BF_cd8s100b	HIST1H2BF	Wald ratio	1	<0.001	2.740 (2.323 − 3.233)
HIST1H2BG_bin	HIST1H2BG	Wald ratio	1	<0.001	2.381 (1.990 − 2.849)
HIST1H2BG_bmem	HIST1H2BG	Wald ratio	1	<0.001	0.548 (0.479 − 0.627)
HIST1H2BG_cd4et	HIST1H2BG	Wald ratio	1	<0.001	2.477 (2.036 − 3.015)
HIST1H2BG_cd8s100b	HIST1H2BG	Wald ratio	1	<0.001	0.338 (0.276 − 0.414)
HIST1H2BI_cd8et	HIST1H2BI	Wald ratio	1	<0.001	0.419 (0.364 − 0.483)
HIST1H2BI_cd8nc	HIST1H2BI	Wald ratio	1	<0.001	2.731 (2.318 − 3.216)
HIST1H2BI_nk	HIST1H2BI	Wald ratio	1	<0.001	0.376 (0.320 − 0.441)
HIST1H2BJ_bin	HIST1H2BJ	Wald ratio	1	<0.001	2.103 (1.831 − 2.416)
HIST1H2BJ_cd8nc	HIST1H2BJ	Wald ratio	1	<0.001	1.518 (1.201 − 1.920)
HIST1H2BL_cd8et	HIST1H2BL	Wald ratio	1	<0.001	0.286 (0.240 − 0.342)
HIST1H2BL_cd8nc	HIST1H2BL	Wald ratio	1	<0.001	0.544 (0.495 − 0.596)
HIST1H2BL_nk	HIST1H2BL	Wald ratio	1	<0.001	2.484 (2.186 − 2.822)
HIST1H2BN_dc	HIST1H2BN	Wald ratio	1	<0.001	0.307 (0.257 − 0.368)
HIST1H2BN_mononc	HIST1H2BN	Wald ratio	1	<0.001	1.469 (1.236 − 1.746)
HIST1H2BN_plasma	HIST1H2BN	Wald ratio	1	<0.001	2.606 (2.216 − 3.063)
HIST1H2BO_cd8et	HIST1H2BO	Wald ratio	1	<0.001	0.693 (0.588 − 0.817)
HIST1H2BO_cd8nc	HIST1H2BO	Wald ratio	1	<0.001	1.333 (1.235 − 1.439)
HIST1H2BO_nk	HIST1H2BO	Wald ratio	1	<0.001	1.885 (1.688 − 2.105)
HIST1H3B_cd4nc	HIST1H3B	Wald ratio	1	<0.001	2.008 (1.732 − 2.329)
HIST1H3D_cd8s100b	HIST1H3D	Wald ratio	1	<0.001	1.847 (1.600 − 2.132)
HIST1H3D_monoc	HIST1H3D	Wald ratio	1	<0.001	0.342 (0.286 − 0.410)
HIST1H3D_mononc	HIST1H3D	Wald ratio	1	<0.001	1.689 (1.393 − 2.049)
HIST1H3H_cd8et	HIST1H3H	Wald ratio	1	<0.001	2.974 (2.504 − 3.533)
HIST1H3H_cd8nc	HIST1H3H	Wald ratio	1	<0.001	3.936 (3.217 − 4.816)
HIST1H3H_nk	HIST1H3H	Wald ratio	1	<0.001	0.658 (0.589 − 0.735)
HIST1H3I_cd8nc	HIST1H3I	Wald ratio	1	<0.001	0.415 (0.365 − 0.472)
HIST1H3J_cd8et	HIST1H3J	Wald ratio	1	<0.001	3.967 (3.257 − 4.832)
HIST1H4A_monoc	HIST1H4A	Wald ratio	1	<0.001	1.945 (1.729 − 2.187)
HIST1H4B_cd4et	HIST1H4B	Wald ratio	1	<0.001	2.769 (2.335 − 3.283)
HIST1H4B_cd8et	HIST1H4B	Wald ratio	1	<0.001	1.972 (1.688 − 2.305)
HIST1H4B_nk	HIST1H4B	Wald ratio	1	<0.001	0.524 (0.457 − 0.602)
HIST1H4E_bmem	HIST1H4E	Wald ratio	1	<0.001	1.815 (1.625 − 2.028)
HIST1H4E_nkr	HIST1H4E	Wald ratio	1	<0.001	1.381 (1.249 − 1.527)
HIST1H4F_cd8et	HIST1H4F	Wald ratio	1	<0.001	0.546 (0.492 − 0.606)
HIST1H4I_bin	HIST1H4I	Wald ratio	1	<0.001	2.757 (2.380 − 3.193)
HIST1H4I_cd4nc	HIST1H4I	Wald ratio	1	<0.001	0.353 (0.302 − 0.412)
HIST1H4I_cd8et	HIST1H4I	Wald ratio	1	<0.001	1.870 (1.708 − 2.047)
HIST1H4J_bmem	HIST1H4J	Wald ratio	1	<0.001	4.378 (3.496 − 5.481)
HIST1H4K_nk	HIST1H4K	Wald ratio	1	<0.001	2.743 (2.208 − 3.407)
HLA−A_cd4nc	HLA−A	Wald ratio	1	<0.001	1.512 (1.360 − 1.682)
HLA−A_cd8s100b	HLA−A	Wald ratio	1	<0.001	1.967 (1.653 − 2.340)
HLA−A_plasma	HLA−A	Wald ratio	1	<0.001	2.065 (1.765 − 2.415)
HLA−B_plasma	HLA−B	Wald ratio	1	<0.001	0.804 (0.729 − 0.886)
HLA−C_cd4et	HLA−C	Wald ratio	1	<0.001	0.807 (0.729 − 0.893)
HLA−C_cd8et	HLA−C	Wald ratio	1	<0.001	1.429 (1.300 − 1.570)
HLA−C_cd8s100b	HLA−C	Wald ratio	1	<0.001	0.772 (0.683 − 0.872)
HLA−C_monoc	HLA−C	Wald ratio	1	<0.001	1.587 (1.450 − 1.737)
HLA−C_mononc	HLA−C	Wald ratio	1	<0.001	1.679 (1.517 − 1.858)
HLA−C_plasma	HLA−C	Wald ratio	1	<0.001	1.998 (1.745 − 2.288)
HLA−DMA_cd4nc	HLA−DMA	Wald ratio	1	<0.001	0.305 (0.270 − 0.346)
HLA−DMA_cd4sox4	HLA−DMA	Wald ratio	1	<0.001	0.619 (0.554 − 0.691)
HLA−DOA_cd8s100b	HLA−DOA	Wald ratio	1	<0.001	3.576 (3.198 − 3.999)
HLA−DOA_nk	HLA−DOA	Wald ratio	1	0.001	0.714 (0.588 − 0.867)
HLA−DOB_cd4nc	HLA−DOB	Wald ratio	1	<0.001	1.323 (1.171 − 1.495)
HLA−DOB_cd8et	HLA−DOB	Wald ratio	1	<0.001	1.447 (1.232 − 1.699)
HLA−DOB_cd8s100b	HLA−DOB	Wald ratio	1	<0.001	0.602 (0.481 − 0.754)
HLA−DPA1_cd4nc	HLA−DPA1	Wald ratio	1	0.002	0.843 (0.757 − 0.938)
HLA−DPA1_cd8et	HLA−DPA1	Wald ratio	1	<0.001	0.791 (0.705 − 0.887)
HLA−DPA1_dc	HLA−DPA1	Wald ratio	1	<0.001	1.321 (1.130 − 1.544)
HLA−DPA1_nk	HLA−DPA1	Wald ratio	1	<0.001	0.773 (0.683 − 0.875)
HLA−DPB1_cd4nc	HLA−DPB1	Wald ratio	1	<0.001	0.662 (0.566 − 0.775)
HLA−DPB1_cd8et	HLA−DPB1	Wald ratio	1	<0.001	0.763 (0.684 − 0.851)
HLA−DQA1_bin	HLA−DQA1	Wald ratio	1	<0.001	0.677 (0.622 − 0.738)
HLA−DQA1_bmem	HLA−DQA1	Wald ratio	1	<0.001	1.439 (1.250 − 1.657)
HLA−DQA1_cd4nc	HLA−DQA1	Wald ratio	1	<0.001	1.901 (1.628 − 2.220)
HLA−DQA1_cd8et	HLA−DQA1	Wald ratio	1	<0.001	0.585 (0.494 − 0.694)
HLA−DQA1_dc	HLA−DQA1	Wald ratio	1	<0.001	1.197 (1.090 − 1.314)
HLA−DQA1_monoc	HLA−DQA1	Wald ratio	1	<0.001	1.591 (1.399 − 1.810)
HLA−DQA1_plasma	HLA−DQA1	Wald ratio	1	<0.001	1.306 (1.164 − 1.464)
HLA−DQA2_cd4et	HLA−DQA2	Wald ratio	1	<0.001	0.481 (0.415 − 0.557)
HLA−DQA2_cd4nc	HLA−DQA2	Wald ratio	1	<0.001	0.545 (0.474 − 0.628)
HLA−DQA2_plasma	HLA−DQA2	Wald ratio	1	<0.001	0.808 (0.765 − 0.854)
HLA−DQB1_bmem	HLA−DQB1	Wald ratio	1	<0.001	0.561 (0.494 − 0.637)
HLA−DQB1_cd4nc	HLA−DQB1	Wald ratio	1	<0.001	1.383 (1.220 − 1.567)
HLA−DQB1_cd4sox4	HLA−DQB1	Wald ratio	1	<0.001	1.869 (1.479 − 2.362)
HLA−DQB1_cd8et	HLA−DQB1	Wald ratio	1	<0.001	2.413 (2.047 − 2.843)
HLA−DQB1_cd8s100b	HLA−DQB1	Wald ratio	1	<0.001	1.860 (1.573 − 2.198)
HLA−DQB1_plasma	HLA−DQB1	Wald ratio	1	<0.001	1.409 (1.233 − 1.609)
HLA−DQB2_cd4nc	HLA−DQB2	Wald ratio	1	<0.001	0.693 (0.573 − 0.838)
HLA−DQB2_cd8et	HLA−DQB2	Wald ratio	1	<0.001	1.372 (1.216 − 1.549)
HLA−DQB2_dc	HLA−DQB2	Wald ratio	1	<0.001	0.478 (0.420 − 0.543)
HLA−DQB2_monoc	HLA−DQB2	Wald ratio	1	<0.001	0.625 (0.559 − 0.699)

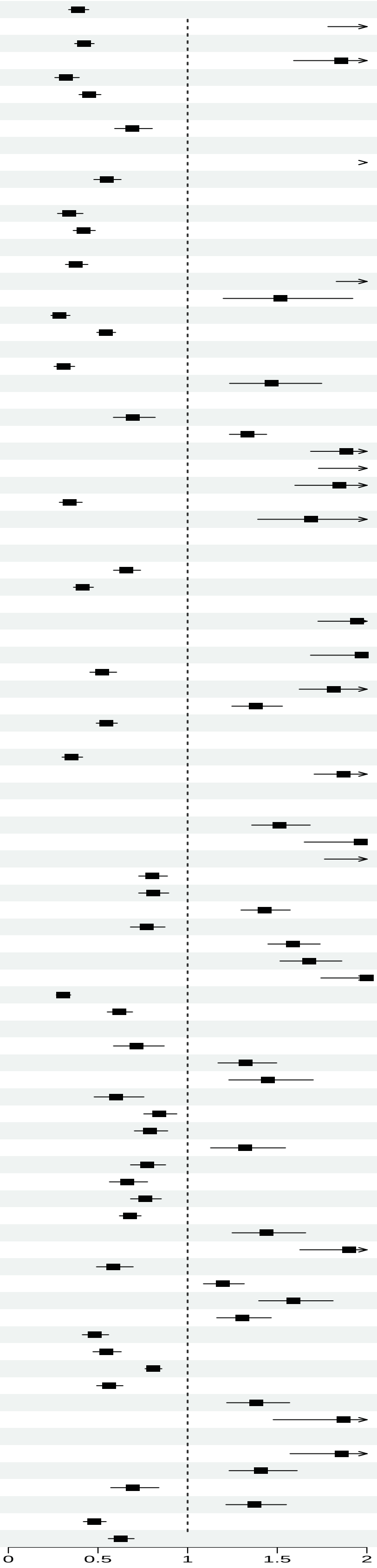

Fig. (S2).

Trait	exposure	Method	nSNP	*P*−Value	OR (95% CI)
HLA−DRB1_bin	HLA−DRB1	Wald ratio	1	<0.001	0.644 (0.607 − 0.684)
HLA−DRB1_bmem	HLA−DRB1	Wald ratio	1	<0.001	0.631 (0.591 − 0.673)
HLA−DRB1_dc	HLA−DRB1	Wald ratio	1	<0.001	0.644 (0.583 − 0.712)
HLA−DRB1_mononc	HLA−DRB1	Wald ratio	1	<0.001	0.459 (0.393 − 0.536)
HLA−DRB1_nk	HLA−DRB1	Wald ratio	1	<0.001	1.593 (1.372 − 1.850)
HLA−DRB1_nkr	HLA−DRB1	Wald ratio	1	<0.001	0.234 (0.198 − 0.276)
HLA−DRB5_cd4nc	HLA−DRB5	Wald ratio	1	<0.001	1.594 (1.460 − 1.741)
HLA−DRB5_cd4sox4	HLA−DRB5	Wald ratio	1	<0.001	1.881 (1.607 − 2.203)
HLA−DRB5_cd8nc	HLA−DRB5	Wald ratio	1	<0.001	1.557 (1.427 − 1.698)
HLA−DRB5_cd8s100b	HLA−DRB5	Wald ratio	1	<0.001	2.290 (1.937 − 2.707)
HLA−E_cd4nc	HLA−E	Wald ratio	1	<0.001	0.726 (0.610 − 0.864)
HMGN4_nkr	HMGN4	Wald ratio	1	<0.001	2.024 (1.820 − 2.250)
HSD17B8_monoc	HSD17B8	Wald ratio	1	<0.001	4.087 (3.628 − 4.603)
HSPA1B_cd4et	HSPA1B	Wald ratio	1	<0.001	1.517 (1.296 − 1.777)
HSPA1B_dc	HSPA1B	Wald ratio	1	<0.001	0.481 (0.440 − 0.525)
IAH1_cd4nc	IAH1	Wald ratio	1	<0.001	0.829 (0.747 − 0.921)
IAH1_cd8nc	IAH1	Wald ratio	1	<0.001	0.709 (0.597 − 0.841)
IER3_bmem	IER3	Wald ratio	1	<0.001	0.431 (0.359 − 0.517)
ITPR3_nk	ITPR3	Wald ratio	1	<0.001	2.140 (1.791 − 2.559)
ITPR3_nkr	ITPR3	Wald ratio	1	<0.001	5.021 (4.389 − 5.744)
ITPR3_plasma	ITPR3	Wald ratio	1	<0.001	0.569 (0.481 − 0.674)
LINC00240_cd4nc	LINC00240	Wald ratio	1	<0.001	1.576 (1.414 − 1.755)
LINC00240_cd8et	LINC00240	Wald ratio	1	<0.001	0.381 (0.330 − 0.440)
LINC00336_cd4nc	LINC00336	Wald ratio	1	<0.001	1.583 (1.390 − 1.802)
LST1_cd4nc	LST1	Wald ratio	1	<0.001	1.304 (1.157 − 1.468)
LST1_mononc	LST1	Wald ratio	1	<0.001	1.313 (1.162 − 1.484)
LY6G5C_bin	LY6G5C	Wald ratio	1	<0.001	2.887 (2.483 − 3.358)
LY6G6F_nk	LY6G6F	Wald ratio	1	<0.001	0.683 (0.591 − 0.789)
MICA_dc	MICA	Wald ratio	1	<0.001	0.454 (0.408 − 0.505)
MICB_cd8et	MICB	Wald ratio	1	<0.001	1.711 (1.429 − 2.049)
MICB_dc	MICB	Wald ratio	1	<0.001	1.427 (1.271 − 1.601)
MICB_nk	MICB	Wald ratio	1	0.001	0.693 (0.561 − 0.856)
MMEL1_cd4nc	MMEL1	Wald ratio	1	<0.001	1.306 (1.157 − 1.474)
MMEL1_mononc	MMEL1	Wald ratio	1	<0.001	1.433 (1.216 − 1.689)
MSH5_bmem	MSH5	Wald ratio	1	<0.001	0.794 (0.705 − 0.893)
MSH5_cd8et	MSH5	Wald ratio	1	<0.001	2.510 (2.161 − 2.916)
NCR3_mononc	NCR3	Wald ratio	1	<0.001	0.488 (0.439 − 0.543)
NFKBIL1_plasma	NFKBIL1	Wald ratio	1	<0.001	2.025 (1.806 − 2.270)
NKAPL_monoc	NKAPL	Wald ratio	1	<0.001	2.404 (2.070 − 2.790)
NOTCH4_cd8et	NOTCH4	Wald ratio	1	<0.001	1.594 (1.314 − 1.934)
NRG1_monoc	NRG1	Wald ratio	1	<0.001	1.248 (1.144 − 1.362)
PBX2_cd4sox4	PBX2	Wald ratio	1	<0.001	1.489 (1.317 − 1.683)
PGBD1_bin	PGBD1	Wald ratio	1	<0.001	2.686 (2.337 − 3.087)
PGBD1_cd8et	PGBD1	Wald ratio	1	<0.001	3.390 (2.841 − 4.046)
PLEKHA1_cd4nc	PLEKHA1	Wald ratio	1	0.001	1.155 (1.065 − 1.253)
PLEKHA1_cd8nc	PLEKHA1	Wald ratio	1	0.001	1.256 (1.105 − 1.429)
PPT2_cd4et	PPT2	Wald ratio	1	<0.001	0.679 (0.592 − 0.779)
PRR3_cd4sox4	PRR3	Wald ratio	1	<0.001	1.902 (1.728 − 2.093)
PRRT1_cd4et	PRRT1	Wald ratio	1	<0.001	0.418 (0.356 − 0.491)
PRSS16_bmem	PRSS16	Wald ratio	1	<0.001	1.942 (1.599 − 2.358)
PRSS16_cd4et	PRSS16	Wald ratio	1	<0.001	2.954 (2.529 − 3.449)
PRSS16_cd8nc	PRSS16	Wald ratio	1	<0.001	3.253 (2.670 − 3.963)
PSMB9_bmem	PSMB9	Wald ratio	1	<0.001	0.488 (0.421 − 0.565)
PSMB9_cd4et	PSMB9	Wald ratio	1	<0.001	0.304 (0.257 − 0.359)
PSMB9_cd4nc	PSMB9	Wald ratio	1	<0.001	0.509 (0.443 − 0.584)
PSMB9_cd8et	PSMB9	Wald ratio	1	<0.001	0.307 (0.261 − 0.361)
PSMB9_cd8nc	PSMB9	Wald ratio	1	<0.001	0.453 (0.385 − 0.533)
PSMB9_mononc	PSMB9	Wald ratio	1	<0.001	0.333 (0.286 − 0.387)
PSMB9_nk	PSMB9	Wald ratio	1	<0.001	0.395 (0.347 − 0.449)
RNASET2_cd4et	RNASET2	Wald ratio	1	<0.001	1.131 (1.062 − 1.204)
RNASET2_cd4nc	RNASET2	Wald ratio	1	<0.001	1.083 (1.040 − 1.127)
RNASET2_cd4sox4	RNASET2	Wald ratio	1	<0.001	1.262 (1.112 − 1.433)
RNASET2_cd8nc	RNASET2	Wald ratio	1	<0.001	1.110 (1.053 − 1.171)
RNASET2_cd8s100b	RNASET2	Wald ratio	1	<0.001	1.197 (1.089 − 1.316)
RNASET2_dc	RNASET2	Wald ratio	1	<0.001	1.402 (1.188 − 1.654)
RNASET2_nk	RNASET2	Wald ratio	1	<0.001	1.159 (1.075 − 1.250)
RNF166_cd4nc	RNF166	Wald ratio	1	0.001	1.220 (1.090 − 1.364)
RP1−265C24.8_nk	RP1−265C24.8	Wald ratio	1	<0.001	0.384 (0.322 − 0.458)
RP1−30M3.5_cd8nc	RP1−30M3.5	Wald ratio	1	0.002	1.266 (1.092 − 1.468)
RP1−30M3.5_nk	RP1−30M3.5	Wald ratio	1	<0.001	1.692 (1.445 − 1.981)
RP11−294J22.6_cd4nc	RP11−294J22.6	Wald ratio	1	0.001	1.351 (1.123 − 1.626)
RP11−294J22.6_cd8et	RP11−294J22.6	Wald ratio	1	0.001	1.506 (1.171 − 1.936)
RP11−294J22.6_nk	RP11−294J22.6	Wald ratio	1	0.001	1.465 (1.158 − 1.855)
RP11−367G6.3_mononc	RP11−367G6.3	Wald ratio	1	<0.001	0.520 (0.452 − 0.598)
RP11−457M11.5_bin	RP11−457M11.5	Wald ratio	1	<0.001	3.083 (2.526 − 3.762)
RP11−457M11.5_cd4et	RP11−457M11.5	Wald ratio	1	<0.001	1.628 (1.375 − 1.928)
RP11−457M11.5_cd8nc	RP11−457M11.5	Wald ratio	1	<0.001	0.688 (0.582 − 0.815)
RP11−457M11.5_cd8s100b	RP11−457M11.5	Wald ratio	1	<0.001	0.409 (0.356 − 0.468)
RP11−514O12.4_nk	RP11−514O12.4	Wald ratio	1	<0.001	0.773 (0.678 − 0.880)
RP11−98G7.1_cd8et	RP11−98G7.1	Wald ratio	1	<0.001	1.337 (1.149 − 1.555)
RP5−1042K10.10_cd8et	RP5−1042K10.10	Wald ratio	1	0.002	0.799 (0.693 − 0.921)
RPP21_dc	RPP21	Wald ratio	1	<0.001	1.766 (1.413 − 2.206)
RTKN2_cd8et	RTKN2	Wald ratio	1	0.002	0.805 (0.700 − 0.925)
RXRB_cd4sox4	RXRB	Wald ratio	1	<0.001	2.018 (1.819 − 2.240)
SCAND3_cd4nc	SCAND3	Wald ratio	1	<0.001	2.086 (1.878 − 2.317)
SESN1_cd4nc	SESN1	Wald ratio	1	<0.001	0.886 (0.833 − 0.942)
SESN1_cd8nc	SESN1	Wald ratio	1	<0.001	0.742 (0.636 − 0.865)
SESN3_cd4nc	SESN3	Wald ratio	1	<0.001	1.269 (1.141 − 1.412)
SESN3_cd8nc	SESN3	Wald ratio	1	<0.001	1.375 (1.193 − 1.586)
SLC12A7_bin	SLC12A7	Wald ratio	1	0.002	0.778 (0.662 − 0.913)
SLC12A7_cd4et	SLC12A7	Wald ratio	1	0.001	1.248 (1.093 − 1.425)

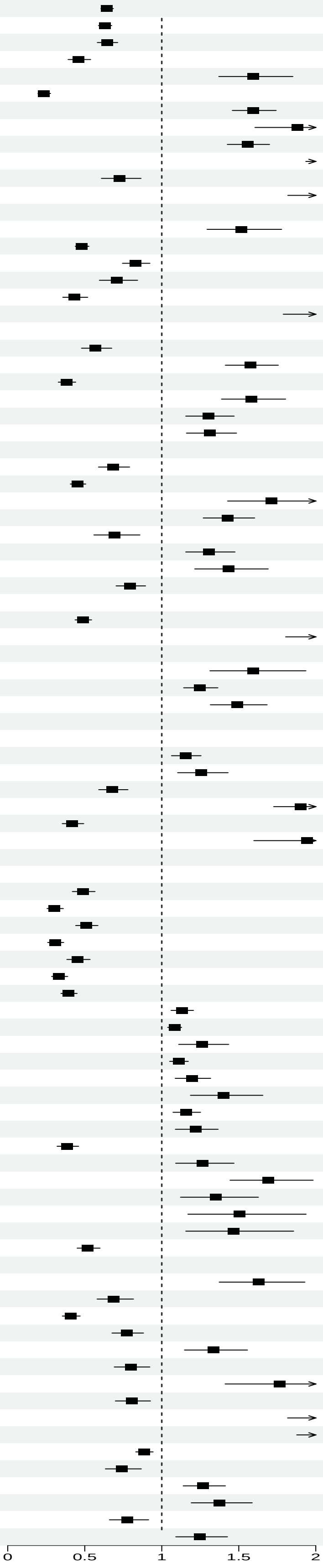

Fig. (S3).

Trait	exposure	Method	nSNP	*P*−Value	OR (95% CI)
SLC12A7_cd4nc	SLC12A7	Wald ratio	1	0.001	1.136 (1.052 − 1.226)
SLC12A7_cd8et	SLC12A7	Wald ratio	1	0.001	1.155 (1.059 − 1.259)
SLC12A7_cd8nc	SLC12A7	Wald ratio	1	0.001	1.173 (1.066 − 1.291)
SLC12A7_monoc	SLC12A7	Wald ratio	1	0.001	1.304 (1.112 − 1.528)
SLC12A7_nk	SLC12A7	Wald ratio	1	0.001	1.115 (1.045 − 1.190)
SLC44A4_nk	SLC44A4	Wald ratio	1	<0.001	0.616 (0.523 − 0.726)
SNAI3_nk	SNAI3	Wald ratio	1	0.001	1.336 (1.129 − 1.580)
TAPBP_nk	TAPBP	Wald ratio	1	0.001	1.371 (1.133 − 1.659)
TCEB3_bmem	TCEB3	Wald ratio	1	0.002	1.285 (1.097 − 1.505)
TCF19_monoc	TCF19	Wald ratio	1	<0.001	0.723 (0.632 − 0.827)
U91328.19_cd4et	U91328.19	Wald ratio	1	<0.001	2.269 (1.976 − 2.605)
UBASH3A_cd4nc	UBASH3A	Wald ratio	1	<0.001	1.386 (1.245 − 1.544)
UBASH3A_cd8nc	UBASH3A	Wald ratio	1	<0.001	1.571 (1.354 − 1.824)
UBE2B_cd4nc	UBE2B	Wald ratio	1	0.001	0.791 (0.688 − 0.910)
UQCC2_cd4nc	UQCC2	Wald ratio	1	0.001	0.800 (0.701 − 0.912)
VARS2_cd8s100b	VARS2	Wald ratio	1	<0.001	0.446 (0.383 − 0.519)
VARS2_mononc	VARS2	Wald ratio	1	<0.001	1.338 (1.208 − 1.482)
VPS52_mononc	VPS52	Wald ratio	1	<0.001	0.417 (0.366 − 0.475)
VPS52_nkr	VPS52	Wald ratio	1	<0.001	0.374 (0.344 − 0.407)
VWA7_cd4nc	VWA7	Wald ratio	1	<0.001	0.579 (0.522 − 0.642)
WBSCR22_cd8et	WBSCR22	Wald ratio	1	0.001	0.740 (0.616 − 0.889)
XXbac−BPG181B23.7_bmem	XXbac−BPG181B23.7	Wald ratio	1	<0.001	0.583 (0.473 − 0.718)
XXbac−BPG181B23.7_cd4et	XXbac−BPG181B23.7	Wald ratio	1	<0.001	0.734 (0.643 − 0.838)
XXbac−BPG181B23.7_cd4nc	XXbac−BPG181B23.7	Wald ratio	1	<0.001	0.783 (0.716 − 0.856)
XXbac−BPG181B23.7_cd8et	XXbac−BPG181B23.7	Wald ratio	1	<0.001	0.636 (0.539 − 0.750)
XXbac−BPG181B23.7_cd8nc	XXbac−BPG181B23.7	Wald ratio	1	<0.001	0.543 (0.452 − 0.651)
XXbac−BPG181B23.7_monoc	XXbac−BPG181B23.7	Wald ratio	1	<0.001	0.604 (0.529 − 0.688)
XXbac−BPG252P9.10_cd4et	XXbac−BPG252P9.10	Wald ratio	1	<0.001	1.263 (1.121 − 1.424)
XXbac−BPG252P9.10_cd8et	XXbac−BPG252P9.10	Wald ratio	1	<0.001	0.484 (0.415 − 0.565)
XXbac−BPG252P9.10_monoc	XXbac−BPG252P9.10	Wald ratio	1	<0.001	0.243 (0.200 − 0.297)
XXbac−BPG252P9.9_cd8et	XXbac−BPG252P9.9	Wald ratio	1	0.002	1.288 (1.099 − 1.509)
XXbac−BPG299F13.17_cd4sox4	XXbac−BPG299F13.17	Wald ratio	1	<0.001	0.674 (0.573 − 0.792)
XXbac−BPG299F13.17_dc	XXbac−BPG299F13.17	Wald ratio	1	<0.001	0.590 (0.501 − 0.695)
XXbac−BPG299F13.17_monoc	XXbac−BPG299F13.17	Wald ratio	1	<0.001	0.283 (0.238 − 0.336)
XXbac−BPG299F13.17_mononc	XXbac−BPG299F13.17	Wald ratio	1	<0.001	0.412 (0.350 − 0.486)
XXbac−BPG308K3.5_cd4nc	XXbac−BPG308K3.5	Wald ratio	1	<0.001	0.629 (0.522 − 0.759)
ZBTB22_dc	ZBTB22	Wald ratio	1	<0.001	3.394 (3.060 − 3.765)
ZBTB22_nkr	ZBTB22	Wald ratio	1	<0.001	1.352 (1.231 − 1.484)
ZBTB22_plasma	ZBTB22	Wald ratio	1	<0.001	2.110 (1.920 − 2.318)
ZBTB9_bin	ZBTB9	Wald ratio	1	<0.001	2.029 (1.728 − 2.383)
ZFP57_cd4et	ZFP57	Wald ratio	1	<0.001	1.649 (1.378 − 1.972)
ZKSCAN3_monoc	ZKSCAN3	Wald ratio	1	<0.001	0.533 (0.462 − 0.615)
ZKSCAN4_bin	ZKSCAN4	Wald ratio	1	<0.001	0.301 (0.253 − 0.358)
ZKSCAN4_cd8s100b	ZKSCAN4	Wald ratio	1	<0.001	2.847 (2.441 − 3.321)
ZKSCAN8_mononc	ZKSCAN8	Wald ratio	1	<0.001	0.270 (0.221 − 0.330)
ZNF165_cd4et	ZNF165	Wald ratio	1	<0.001	3.067 (2.535 − 3.712)
ZNF322_dc	ZNF322	Wald ratio	1	<0.001	2.619 (2.267 − 3.025)
ZNF391_cd4et	ZNF391	Wald ratio	1	<0.001	2.014 (1.822 − 2.227)
ZNF786_cd4nc	ZNF786	Wald ratio	1	<0.001	1.259 (1.119 − 1.417)
ZNF786_cd8et	ZNF786	Wald ratio	1	0.001	0.740 (0.623 − 0.879)
ZNF786_cd8nc	ZNF786	Wald ratio	1	<0.001	1.433 (1.191 − 1.726)
ZSCAN12_cd8s100b	ZSCAN12	Wald ratio	1	<0.001	0.470 (0.399 − 0.552)
ZSCAN16_nkr	ZSCAN16	Wald ratio	1	<0.001	3.982 (3.267 − 4.853)
ZSCAN31_cd8nc	ZSCAN31	Wald ratio	1	<0.001	3.220 (2.728 − 3.802)
ZSCAN9_cd8nc	ZSCAN9	Wald ratio	1	<0.001	0.461 (0.401 − 0.530)
ABHD16A_bin	ABHD16A	IVW	3	<0.001	1.736 (1.592 − 1.893)
ATP6V1G2_cd8s100b	ATP6V1G2	IVW	2	<0.001	1.927 (1.342 − 2.768)
C2_cd8nc	C2	IVW	4	<0.001	1.473 (1.210 − 1.794)
CCHCR1_dc	CCHCR1	IVW	3	<0.001	0.710 (0.601 − 0.839)
CCHCR1_monoc	CCHCR1	IVW	2	0.001	1.170 (1.063 − 1.288)
CCHCR1_nkr	CCHCR1	IVW	3	0.001	0.522 (0.358 − 0.761)
DDX39B−AS1_cd4nc	DDX39B−AS1	IVW	2	<0.001	0.619 (0.517 − 0.742)
DHX16_cd4sox4	DHX16	IVW	3	<0.001	1.443 (1.338 − 1.557)
EGFL8_nk	EGFL8	IVW	2	<0.001	0.531 (0.476 − 0.593)
FES_nk	FES	IVW	2	0.001	1.106 (1.040 − 1.177)
FKBPL_mononc	FKBPL	IVW	2	<0.001	2.011 (1.776 − 2.277)
HCG25_cd4et	HCG25	IVW	2	<0.001	2.161 (1.495 − 3.124)
HLA−A_bin	HLA−A	IVW	2	<0.001	0.629 (0.579 − 0.683)
HLA−DQA1_nkr	HLA−DQA1	IVW	2	<0.001	0.532 (0.480 − 0.590)
HLA−DQA2_monoc	HLA−DQA2	IVW	3	<0.001	0.820 (0.746 − 0.902)
HLA−DQB2_cd8nc	HLA−DQB2	IVW	2	<0.001	0.544 (0.432 − 0.685)
HLA−G_mononc	HLA−G	IVW	2	0.001	0.412 (0.242 − 0.702)
HSPA1B_nkr	HSPA1B	IVW	2	<0.001	0.450 (0.325 − 0.622)
HSPA1L_monoc	HSPA1L	IVW	2	<0.001	1.420 (1.233 − 1.635)
LTA_cd4sox4	LTA	IVW	5	<0.001	1.630 (1.344 − 1.978)
LY6G5C_mononc	LY6G5C	IVW	2	<0.001	1.532 (1.410 − 1.665)
MICA_nkr	MICA	IVW	3	<0.001	0.563 (0.417 − 0.759)
POU5F1_nk	POU5F1	IVW	3	<0.001	0.612 (0.502 − 0.746)
RP11−973H7.1_nk	RP11−973H7.1	IVW	2	0.001	0.872 (0.802 − 0.949)
RPP21_cd4sox4	RPP21	IVW	3	<0.001	0.743 (0.640 − 0.863)
TRIM39_dc	TRIM39	IVW	3	<0.001	1.743 (1.364 − 2.227)
XXbac−BPG249D20.9_cd4nc	XXbac−BPG249D20.9	IVW	2	<0.001	0.739 (0.676 − 0.809)
XXbac−BPG252P9.10_bin	XXbac−BPG252P9.10	IVW	2	<0.001	1.550 (1.244 − 1.930)
XXbac−BPG252P9.9_cd8nc	XXbac−BPG252P9.9	IVW	2	<0.001	2.222 (1.646 − 2.999)
ZBTB9_bmem	ZBTB9	IVW	2	<0.001	2.374 (1.535 − 3.674)
ZFP57_bmem	ZFP57	IVW	3	<0.001	1.519 (1.424 − 1.621)
ZFP57_cd4nc	ZFP57	IVW	2	<0.001	1.542 (1.418 − 1.677)
ZKSCAN3_cd8s100b	ZKSCAN3	IVW	2	<0.001	0.503 (0.374 − 0.675)
ZKSCAN4_monoc	ZKSCAN4	IVW	2	<0.001	0.526 (0.424 − 0.654)

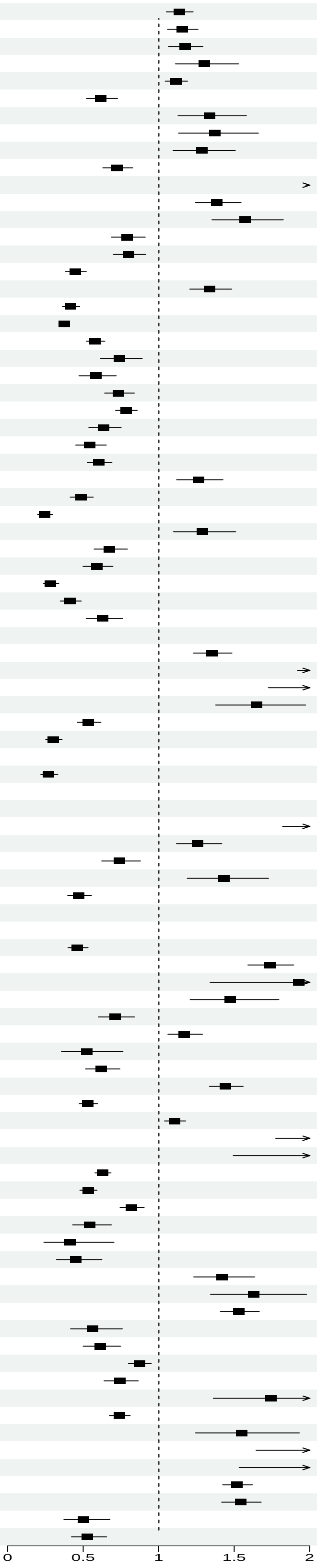

Fig. (S4).
